# Current Status of Cancer Care for Young Patients with Nasopharyngeal Carcinoma in Jakarta, Indonesia

**DOI:** 10.1371/journal.pone.0102353

**Published:** 2014-07-14

**Authors:** Marlinda Adham, Sharon D. Stoker, Maarten A. Wildeman, Lisnawati Rachmadi, Soehartati Gondhowiardjo, Djumhana Atmakusumah, Djayadiman Gatot, Renske Fles, Astrid E. Greijer, Bambang Hermani, Jaap M. Middeldorp, I. Bing Tan

**Affiliations:** 1 Ear, Nose and Throat, University of Indonesia, Dr. Cipto Mangunkusumo hospital, Jakarta, Indonesia; 2 Anatomy-Pathology, University of Indonesia, Dr. Cipto Mangunkusumo hospital, Jakarta, Indonesia; 3 Radiotherapy, University of Indonesia, Dr. Cipto Mangunkusumo hospital, Jakarta, Indonesia; 4 Haematology-Medical Oncology Internal Medicine, University of Indonesia, Dr. Cipto Mangunkusumo hospital, Jakarta, Indonesia; 5 Medical Oncology Pediatric Department, University of Indonesia, Dr. Cipto Mangunkusumo hospital, Jakarta, Indonesia; 6 Department of Head and Neck Oncology and Surgery, The Netherlands Cancer Institute, Amsterdam, The Netherlands; 7 Department of Pathology, VU University Medical Center, Amsterdam, The Netherlands; 8 Ear, Nose and Throat Department, Gadjah Mada University, Yogyakarta, Indonesia; 9 Department of Otorhinolaryngology, Academic Medical Centre, Amsterdam, The Netherlands; 10 Department of Oral and Maxillofacial Surgery, Academic Medical Centre, Amsterdam, The Netherlands; Karolinska Institutet, Sweden

## Abstract

**Background:**

Nasopharyngeal carcinoma (NPC) is endemic in Indonesia and 20% of the patients are diagnosed before the age of 31. This study evaluates presentation and treatment outcome of young patients in Jakarta, in a tertiary referral centre.

**Methods:**

Forty-nine patients under the age of 31, diagnosed with NPC between July 2004 and January 2007, were evaluated. Baseline data included histological type, stage of disease and presenting symptoms. We intended to follow all patients after diagnosis to reveal treatment outcome and overall survival (OS).

**Results:**

All but two patients had advanced stage disease (94%), 7 (14%) had distant metastasis. The median interval between start of complaints and diagnosis was 9 months. Forty-two patients were planned for curative intent treatment. Eleven patients (26%) never started treatment, 2 patients did not complete treatment and 3 patients did not return after finishing treatment. Four patients died before radiation could start. Three patients died within 4 months after treatment. Nine patients (21%) had a complete response. Due to the high number of patients who were lost to follow-up (LFU), OS was analyzed as follows: a best-case (patients censored at last contact) and a worst-case scenario (assuming that patients who did not finish treatment or had disease at last contact would have died). The 2-year OS for patients without distant metastases was 39–71%.

**Conclusion:**

Treatment outcome for young patients with NPC in this institute was poor. Improvement can be achieved when NPC is diagnosed at an earlier stage and when there is better treatment compliance.

## Introduction

The incidence of nasopharyngeal carcinoma (NPC) in Indonesia is estimated to be 6∶100.000, meaning that every month at least 1000 patients are diagnosed. Probably related to better diagnostics and improved awareness this number increases every year [1]. In Jakarta 20% of the patients are diagnosed before the age of 31 [1]. A study conducted in Yogyakarta, revealed a 3-year overall survival of 30% for adults with NPC, compared to 80% in literature [2], [3]. The current study reveals the presentation and treatment outcome of young patients in Jakarta, in a tertiary referral centre.

NPC in young patients differs in certain aspects from adults. The percentage of non-keratinizing undifferentiated carcinoma is higher, and the association with Epstein-Barr virus (EBV) is stronger [1], [4]–[6]. Young patients have more advanced disease at diagnosis and distant metastases are more frequently seen [4]–[9]. This might be caused by the undifferentiated state of the tumor, which is prone to develop distant metastasis [4], [6]–[7]. Another hypothesis is the late recognition of complaints belonging to NPC in young patients, since early symptoms of NPC are non-specific and can look like ordinary upper airway infections, which are common in children.

Treatment for young patients generally follows the guidelines established for adults; radiotherapy on the nasopharynx and cervical nodal levels, usually combined with chemotherapy [4]. Despite the advanced stage at presentation, survival of young patients does not differ from adults. Several retrospective trials have proven the benefit of additional chemotherapy in juveniles [4], [9]–[12]. Five-year disease-free survival varies between 45–77% and 5-year overall survival is 52–77% [5], [7]–[12]. Recently, Buehren et al. published more promising results by adding adjuvant interferon beta after standard (chemo-) radiotherapy. This resulted in an event-free survival rate of 92.4% after a median follow-up of 30 months and an overall survival of 97.1% [7], [13].

All these results are derived from top-end hospitals and some are in clinical trial settings. Here we present a prospective observational study on routine treatment results of young patients with NPC at a top end hospital in Jakarta. We will describe the tumor characteristics and complaints at presentation, the given treatment and the treatment outcome.

## Methods

### Patients

This was a prospective cohort study. All patients diagnosed with NPC between July 2004 and January 2007 at the Rumah Sakit Cipto Mangunkusumo (RSCM), a university hospital in Jakarta, were eligible for inclusion. Patients were included if they were below the age of 31 at diagnosis and had histological proven NPC. In this period 228 patients were diagnosed with NPC, and 49 patients met the inclusion criteria of age and histological confirmed NPC. Ethical approval was obtained at the Ethical committee of the Faculty of Medicine of the university of Indonesia. All patients or their parents/ legal guardian signed informed consent. To get more insight in the specific problems of patients at young age, the patients were divided into two groups, i.e. ≤15 years and >15–30 years.

Baseline information consisted of patient demographics, including the type of insurance. Jakarta has three types of insurances; jamkesmas (poor people), askes (civil servants) and patients who pay health care out of the pocket or have private insurance (self-finance) [14]–[15]. We hypothesized that type of insurance would have an impact on treatment outcome.

### Presentation and Diagnosis

Information on symptoms was gathered from the clinical medical record. Symptoms were scored for presence at diagnosis and duration till diagnosis. The histological diagnosis was made according to the World Health Organization (WHO) classification, WHO type 1; keratinizing squamous cell carcinoma, WHO type 2; non-keratinizing squamous cell carcinoma, WHO type 3; undifferentiated carcinoma. The extent of disease was determined by clinical examination using rigid or flexible nasopharyngoscopy, Computed Tomography (CT)-scan of the head and neck region, chest radiography, ultrasonography of the abdomen and a bone scan. Tumor stage was classified according to the 2002 criteria of the 6th American Joint Committee on Cancer (AJCC).

### Treatment

Due to the waiting time to radiation, different schedules were used. Three different radiation schedules were used; conventional fractionated schedule (daily fraction of 2 Gy, total 33–35 fractions); hyper fractionated schedule (2 fractions/ day of 1.2 Gy with 6 hours in between, total dose 81.6 Gy); accelerated hyper fractionated schedule (daily fraction of 1.8 Gy in the first 4 weeks, followed by 2 weeks of daily 1 fraction of 1.8 Gy and a surdosage to macroscopic tumor of 1.5 Gy with 6 hours in between, total 72 Gy). All schedules could be completed in 6–7 weeks. Neo-adjuvant chemotherapy consisted of intravenous cisplatin 100 mg/m2/day on day 1, and 5-fluoro-uracil 1000 mg/m2/day on day 1–5, every 3 weeks for 3–4 courses. Concurrent chemotherapy consisted of intravenous cisplatin 40 mg/m2 weekly during radiotherapy.

Patients with distant metastasis received palliative chemotherapy; cisplatin 100 mg/m2/day on day 1 and, 5-fluoro-urasil 1000 mg/m2/day on day 1–4. The number of courses depended on the clinical condition. Palliative radiotherapy was given on bone metastasis.

### Follow-up

Patients were scheduled for routine follow-up at the outpatient clinic. Treatment response measurements were planned 8 to 12 weeks after treatment, by physical examination, nasopharyngoscopy and CT-scan. The follow-up schedule proceeds with 3 monthly visits during the first 2 years after radiotherapy.

### Statistics

To test for association between age and tumor stage at diagnosis, linear-by-linear test was used. For symptoms at diagnosis two scales were constructed: the number of complaints at diagnosis and the maximum duration to diagnosis. For patient's missing data on symptom duration, the median duration was imputed. Associations between these two scales and both age (as a continuous variable) and AJCC stage were tested using linear-by-linear tests.

Association between age and diagnosis-to-treatment interval (DTI) and overall-radiotherapy-treatment time (OTT) was assessed by Spearman correlation test. The Statistical Package for the Social Sciences, version 20 was used for analysis. P-values less than 0.05 were considered as significant.

Kaplan-Meier analyzed overall survival. Survival time was defined as the time between the date of diagnosis till the date of death. Stratification was done by M stage, and M0 was further stratified by age (0–15 and 16–30). For comparison between the 0–15 and the 16–30 age group a log rank test was used.

## Results

### Patients

Forty-nine patients were included. The median age was 21 and ranged between 3–30 years. WHO type 3 was the histological type in 46 patients (94%) and 3 patients (6%) had WHO type 1. The mean follow-up period for the patients without distant metastasis at diagnosis was 18 months and for patients with distant metastasis 7 months.

### Stage of disease at presentation

T-stage was dominated by advanced stage (66%). Lymph node metastasis was seen in 96% of the patients (table 1). Ninety-four per cent had advanced stage of disease. Seven patients (14%) had distant metastasis at diagnosis. All had metastases to the bone. In addition, two patients had lung metastasis and one of these had also liver metastasis. No association was found between age and stage of disease at presentation (linear-by-linear p = 0.85).

**Table 1:** Patient & tumor characteristics.

		0–15 Year	16–30 Year
		n = 14	n = 35
**AGE AT DIAGNOSIS**			
	Median	11	26
	(Range)	(3–15)	(16–30)
**GENDER**		Number (percentage)	Number (percentage)
	Male	10 (71)	18 (51)
	Female	4 (29)	17 (49)
**INSURANCE**			
	Jamkesmas	14 (100)	27 (77)
	Askes	0 (0)	2 (6)
	Self Finance	0 (0)	4 (11)
	Missing		2 (6)
**TUMOR**			
T	T1	0 (0)	2 (6)
	T2a	0 (0)	2 (6)
	T2b	4 (29)	9 (26)
	T3	5 (36)	8 (23)
	T4	5 (36)	14 (40)
**N**	N0	0 (0)	2 (6)
	N1	0 (0)	7 (20)
	N2	3 (21)	7 (20)
	N3a	8 (57)	15 (43)
	N3b	3 (21)	4 (11)
**M**	M0	12 (86)	30 (86)
	M1	2 (14)	5 (14)
**STAGE**	2b	0 (0)	3 (9)
	3	3 (21)	4 (11)
	4a	0 (7)	9 (26)
	4b	9 (64)	14 (40)
	4c	2 (14)	5 (14)

### Symptoms at diagnosis

Information on presenting symptoms at diagnosis was available for 41 patients (table 2). The median number of complaints at diagnosis was 5 (range 2–10). The median interval to diagnosis was 9 months (range 1–36 months). A neck mass was mentioned in 93% of the patients at diagnosis, more than 50% of the patients (21/40) had bilateral neck masses.

**Table 2:** Complaints at diagnosis and interval between first appearance and diagnosis.

	0–15 Year	16–30 Year		0–15 Year	16–30 Year
	n = 10 (100%)	n = 31 (100%)	Duration of symptom in months		
**NECK MASS**					
Yes	10 (100)	28 (90)	Median	11	9
No	0 (0)	2 (6)	Range	5–18	2–36
Missing		1 (3)			
**NASAL CONGESTION**					
Yes	8 (80)	23 (74)	Median	4	3
No	2 (20)	8 (26)	Range	1–18	1–12
			Missing		1
**EPISTAXIS**					
Yes	7 (70)	17 (55)	Median	4	2
No	3 (30)	14 (45)	Range	1–18	1–12
			Missing		1
**POST NASAL DRIP**					
Yes	4 (40)	10 (32)	Median	7	10
No	6 (60)	17 (55)	Range	4–10	3–12
Missing		4 (13)	Missing	2	4
**DIPLOPIA**					
Yes	3 (30)	9 (29)	Median	1	3
No	7 (70)	22 (71)	Range	0.5–5	0.25–7
**DEAFNESS**					
Yes	7 (70)	21 (68)	Median	1	1
No	3 (30)	10 (32)	Range	1–2	1–2
			Missing		1
**TINNITUS**					
Yes	5 (50)	17 (54)	Median	3	3
No	5 (50)	14 (45)	Range	2–12	1–24
			Missing		2
**EAR PAIN**					
Yes	2 (20)	7 (23)	Median	2	1.5
No	6 (60)	20 (65)	Range	2–2	0.03–12
Missing	2 (20)	4 (13)	Missing	1	3
**OTORRHEA**					
Yes	0 (0)	3 (10)	Median		6
No	9 (90)	22 (71)	Range		6–12
Missing	1 (10)	6 (19)			
**CEPHALGIA**					
Yes	5 (50)	21 (68)	Median	6	3
No	5 (50)	10 (32)	Range	1–12	1–36
			Missing		2
**NERVE PARALYSIS**					
Yes	2 (20)	5 (16)	Median	5.5	1
No	7 (70)	25 (81)	Range	5–6	1–5
Missing	1 (10)	1 (3)	Missing		1

No associations (linear-by-linear) were detected between either age and the number of complains at diagnosis (p = 0.41) or the duration to diagnosis (p = 0.79). Also no associations were found between the stage of disease at diagnosis and the number of complaints (p = 0.25) and interval to diagnosis (p = 0.29).

### Treatment

Forty-two patients could be planned for treatment with curative intent. For 22 patients data was available on given radiotherapy treatment (table 3). The median interval between diagnosis and radiotherapy was 110 days (28–690 days). Patients in the 16–30 age group had to wait longer than the younger patients (130 vs. 77 days), although no association with age was found (Spearman correlation p = 0.99).

**Table 3:** Radiotherapy treatment.

	0–15 Year	16–30 Year
**DIAGNOSIS TO RADIOTHERAPY IN DAYS**	n = 7	n = 15
Median	77	130
(Range)	(28–690)	(34–320)
**RADIOTHERAPY DURATION IN DAYS OVERALL**	n = 4	n = 14
Median	55	56
(Range)	(50–160)	(38–77)

For 18 patients data on the overall radiotherapy treatment time (OTT) was available. The median OTT was 55 days (range 38–160), no association with age was found (spearman correlation p = 0.41). Since almost all patients had jamkesmas insurance, no association between the insurance type and the DTI or OTT could be found.

### Treatment outcome and follow-up

**Figure 1:**
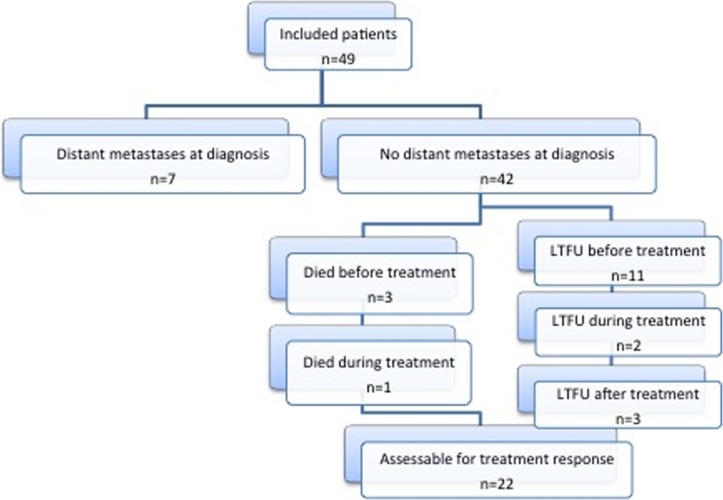
Patient overview. LTFU =  lost to follow-up.

Directly after diagnosis 11 patients (26%) did not return to the hospital. Two patients stopped therapy during treatment, and directly after therapy 3 patients never returned to the hospital. Despite several attempts to contact these 16 patients, no information on their health status could be retrieved. Four patients died before radiation treatment could start. Figure 1 shows the chart-flow.

Accordingly, for 22 patients the effect of treatment could be studied. Three patients died within 4 months after radiotherapy, before response was assessed. For three patients survival data was available, but no data on therapy response; one died 19 months after neo-adjuvant chemotherapy (unknown if he finished radiation treatment), one was alive 30 months after radiation and clinical suspect for distant metastases, and one died 65 months after radiation (reason unknown).

Nine patients underwent examination 2–3 months after treatment, one patient had the examination 1 month after treatment and six patients had examination later than 3 months after therapy. Complete response was seen in 9 of these 16 patients, partial response in 5 patients and progressive disease in 2 patients.

### Overall survival

The number of patients who were lost to follow-up (LFU) in this study was high. Despite multiple attempts to contact them or their family, it was not possible to minimize the missing data. The assumption that the risk to death was equally distributed between patients who were LFU and patients who were still in the study is not likely. This is based on the fact that some patients never started treatment, stopped during treatment or had disease at the last date of follow-up. Therefore we made two Kaplan-Meier curves, representing a best-case scenario and a worst-case scenario. The best-case scenario is a regular Kaplan-Meier curve, wherein all patients are censored on the last date of follow-up. For the worst-case scenario; for patients without distant metastasis, all patients who did not return to the hospital before starting treatment (n = 11), before finishing treatment (n = 2), or who had disease at last moment of contact (n = 6) were assumed to be death at the last date of contact; for patients with distant metastasis at diagnosis, the last date of contact was set as the date of death. A realistic overall survival curve will be positioned between these two Kaplan-Meier curves.

**Figure 2:**
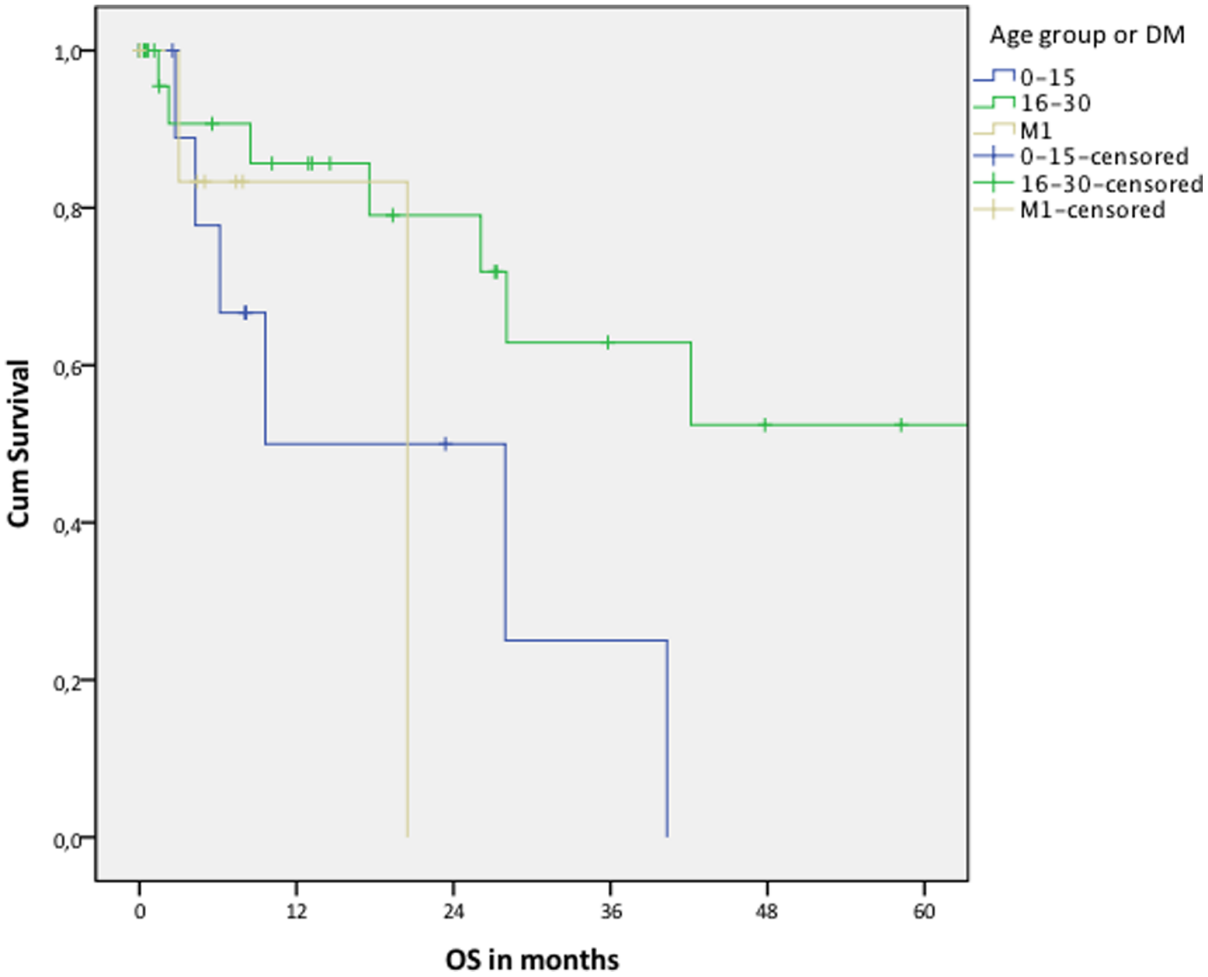
Overall survival: best-case scenario. All patients who were lost to follow up were censored at the moment of last contact (Log rank is p = 0.021, when comparing patients without distant metastasis: 0–15 vs. 16–30 year). DM  =  distant metastasis at diagnosis; OS  =  overall survival.

**Figure 3:**
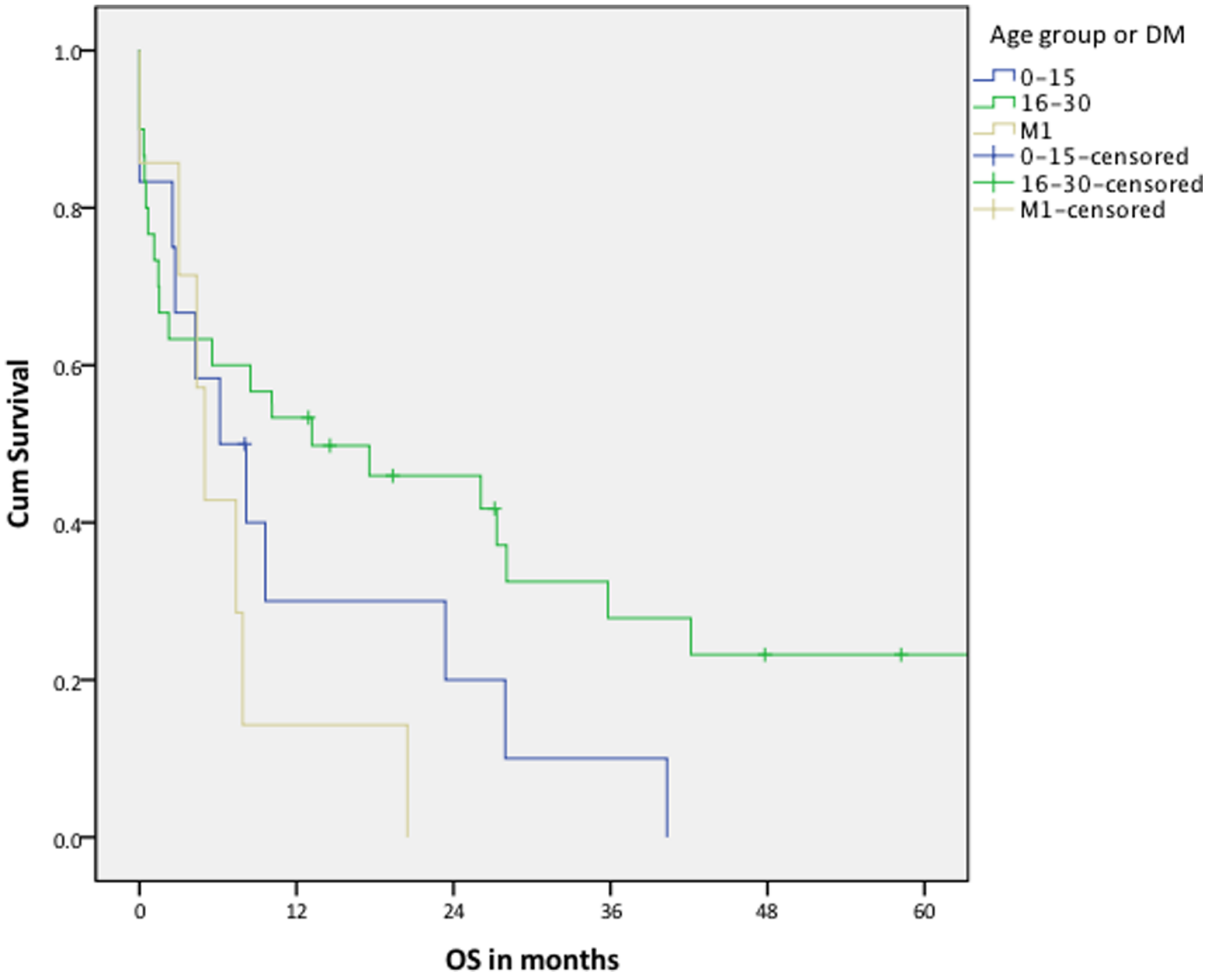
Overall survival: worst-case scenario. All patients who were lost to follow up before treatment (n = 11) or during treatment (n = 2), or who had disease at last moment of follow up (n = 6) are assumed to be death (Log rank p =  0. 142, when comparing patients without distant metastasis: 0–15 vs. 16–30 year). DM  =  distant metastasis at diagnosis; OS  =  overall survival.

The 2-year overall survival for patients without distant metastasis at diagnosis was 39–71% (worst- and best-case scenario, respectively). The 2-year survival for patients with distant metastasis at diagnosis was 0% (table 4). Overall survival, analyzed in the best-case scenario was significantly poorer for the younger patients (log rank p = 0.021). In the worst-case scenario this was not significant (log rank p = 0.142)(figure 2 and 3).

**Table 4:** Overall survival.

	6 months		2 years		5 years	
	Worst-case scenario	Best-case scenario	Worst-case scenario	Best-case scenario	Worst-case scenario	Best-case scenario
**0–15 year (M0, n = 12)**	58%	78%	20%	50%	0%	0%
**16–30 year (M0, n = 30)**	60%	91%	46%	79%	23%	52%
**0–30 year (M0, n = 42)**	60%	87%	39%	71%	16%	38%
**Distant metastasis (n = 7)**	43%	83%	0%	0%	0%	0%

Overall survival was tested on association with stage of disease, symptoms at diagnosis, waiting time for radiotherapy and treatment duration. No significant results were found. It was not possible to test for association with insurance, since the group of patients with other insurance than jamkesmas was too small.

## Discussion

Cancer is the leading cause of death worldwide [16]–[17]. The distribution of cancer mortality shifts towards the low- and middle-income countries. Currently, 70% of cancer deaths occur in these countries and this burden increases every year [16]–[18]. Their health-care systems are not prepared for the number of patients. Unlike high-income countries, where cancer survival improves due to better treatment facilities and enhanced protocols, low-income countries lack facilities and medication. Funding for research and solutions aiming to resolve these limitations is hardly available. The gap in treatment results between high-income and low-income countries is therefore widening [17]. Major improvement in the health care systems is needed. Although many authors have emphasized this, solid data on the actual problems are lacking. This study reveals some of the current problems in the treatment of NPC in Jakarta, a major referral hospital and one of the top end hospitals in Indonesia.

Cancer care for young patients with NPC in Jakarta is poor compared to international literature. In literature, 1–4% of the young patients with NPC have distant metastasis at initial diagnosis [4]–[5]. In this study 14% of the patients presented with distant metastasis. Two years after diagnosis many patients were lost to follow-up, only 47 per cent of them were still in the study (23/49). Ten out of these 23 patients had already died at this point. The 5-year overall survival for patients without distant metastasis lies between 16–38%, compared to 52–77% in the literature [4]–[5], [7]–[13]. These results might be caused by the late stage of presentation at the hospital, insufficient treatment (compliance) and poor follow-up.

Advanced stage of disease at diagnosis was seen in 94% of the patients. Since stage of disease is strongly associated with prognosis, this partly accounts for the poor survival. Advanced stage at diagnosis is related to a long interval to diagnosis [19]. In our study the mean interval from start of complaints till diagnosis was 9 months, which is long compared to the 4 to 8 months found in China [19], India [12] and Turkey [5]. This long interval can be caused both by patient's or doctor's delay. Early stage symptoms of NPC look like an ordinary inflammatory upper airway infection. In our young patient group, the early stage symptoms are not mentioned as complaints with the longest duration. Apparently, the early symptoms are not evidently present or do not trigger patients to seek medical help. The latter explanation might be plausible in this patient group, due to the non-specificity of the complaints and the frequency of upper airway complaints in the young population.

Neck masses, a late stage symptom, were present in 93 per cent of our patients at diagnosis. In almost all patients this complaint existed with the longest interval to diagnosis. One should assume that when a neck mass is present, a patient (or parent) should make effort to consult a doctor. Instead, a time interval of 9.5 months was found before definitive diagnosis. It seems that patients (and probably doctors) are not aware of the probability for NPC involvement in young patients with an unexplained neck mass.

Patient's delay to diagnosis can also be caused by the long distance to health care facilities or limited financial resources of patients, 84% had poor men's insurance. Besides, we know by experience that many patients first seek medical help in the alternative circuit. Even when NPC is diagnosed some patients prefer alternative therapy above conventional. We cannot confirm this by our study results, but eleven patients did not return to the hospital after diagnosis. Unfortunately, we could not retrieve the reason for not returning. More public awareness about the symptoms of NPC and need for early treatment with (chemo-) radiotherapy can contribute to an earlier consultation of the doctor and better compliance to the advised therapy. Previous studies have already shown the effectiveness of public awareness campaigns in breast and cervical cancer [20].

As mentioned before, the doctor can also cause the delay to diagnosis; when doctors do not recognize the symptoms as related to cancer or when they are not aware of the high probability of NPC. Earlier research revealed that the knowledge of general practitioners (GP's) on NPC in Indonesia was insufficient [21]. A sequel study showed that after teaching there was a great improvement of knowledge [22]. More educational programs can improve early diagnosis. Furthermore, with the increasing awareness of NPC's associated with Epstein-Barr virus (EBV) infection and the availability of tests with EBV-related tumor markers which can be performed by the GPs, improvement in earlier diagnosis is within reach [1], [23]–[25].

Another result of this study was the insufficiency of the treatment itself. The median interval between diagnosis and radiotherapy was almost 4 months. This is partly caused by the insurance system. Almost all patients had jamkesmas insurance. Hereby, approval is needed for every investigation and treatment, which takes valuable time. Another key reason for the long interval to treatment is shortage in capacity of radiotherapy facilities. In 2008, 35 radiotherapy devices were available for a population of 229 million. A substantial number of these devices are out of order on a regular base, resulting in 0.13 accelerators per million inhabitants [26]. This is not enough. For comparison, in Europe 2–5.5 accelerators are available per one million inhabitants [27]. We assumed that the patient's type of insurance would have a strong impact on all parameters, unfortunately no statistical analysis could be performed due to the small group who had other insurance than Jamkesmas. The presented results do emphasize the low financial resources of this patient group and the need for improvement of the national health care system.

The long waiting time, the neo-adjuvant chemotherapy to overcome the waiting time and the treatment of radiation (with or without concurrent chemotherapy) has impact on patient's physical status. In this study three patients died before treatment could start, one patient died during neo-adjuvant treatment and another four patients died directly after treatment, accordingly 19% died soon after diagnosis. This percentage might be an underestimation, since directly after diagnosis 11 patients were lost to follow-up. These patients did not get treatment, so it is assumable that some of them also would have died. The results are comparable to a recent study of adults with NPC, conducted in Yogyakarta, here 13% of the patients died before radiotherapy started and 29% died before treatment response could be assessed [2]. Studies involving preservation or improving the patient's physical performance status during the waiting time and during treatment might be of great value to lower the mortality. Suggestions are other treatment modalities, like photodynamic therapy to overcome the waiting time, or protocols to observe and improve the nutritional status [28]

The overall treatment time of radiotherapy was 55 days. Optimally, a total dose of 66 to 70 Gray should be given in 33–35 fractions in a maximum of 47 days. For each day by which radiotherapy treatment is extended, effective dose is lost, and the success rate declines rapidly [29]–[30]. The long overall treatment time is therefore most probably also a reason for the poor complete response percentage.

Another problem that we encountered was the lack of data management and poor follow-up. This made it impossible to compare the different treatment protocols and made statistical analysis difficult. In general the lack of proper data management causes a lack of essential feedback for doctors, which results in the absence of a learning curve and current insight in problems in cancer care in general. Besides, poor follow-up results in late recognition of recurrent disease, which immediately affects the patient's health and chances of survival. A digital data management system may result in better insights in clinical performance and stimulate the treatment learning curve [31].

## Conclusions

This is the first study presenting the treatment results of young patients with NPC in Indonesia, where 20% of the patients are diagnosed before the age of 31. Comparable, poor treatment outcome has been found in an independent study among adults with NPC in Yogyakarta, and it is assumable that other low and middle-income countries are coping with similar problems in handling NPC patients [2], [28]. The study revealed serious weaknesses at different levels in diagnosis and treatment. The current changes in the insurance system of Indonesia, aiming to provide health care for every one, will put even more pressure on the health care facilities. Therefore it is likely that the problems might get bigger.

Establishing more radiotherapy facilities would be the best step to solve a big part of the problems. However, even when financial resources are not the limiting factor, it will take a decade to built new bunkers and educate doctors and nurses to accomplish this. In the meanwhile the focus should be to treat people who can have treatment in a proper way. Earlier diagnosis, better treatment compliance and improved follow-up are the key points to accomplish this. More public, medical and patient awareness for these key points might be one of the answers.
